# Complement component C3 is associated with body composition parameters and sarcopenia in community-dwelling older adults: a cross-sectional study in Japan

**DOI:** 10.1186/s12877-024-04720-z

**Published:** 2024-01-27

**Authors:** Misa Nakamura, Masakazu Imaoka, Keiko Sakai, Takanari Kubo, Ryota Imai, Mitsumasa Hida, Fumie Tazaki, Junya Orui, Takao Inoue, Masatoshi Takeda

**Affiliations:** Department of Rehabilitation, Osaka Kawasaki Rehabilitation University, 158 Mizuma, Kaizuka, Osaka, 597-0104 Japan

**Keywords:** Sarcopenia, Complement, C3, Body composition, Older adults

## Abstract

**Background:**

Chronic inflammation is a factor in the pathogenesis of sarcopenia, which is characterized by low muscle mass and reduced strength. Complement C3 is important in the management of the immune network system. This study seeks to determine the relationship between serum C3 levels and body composition and sarcopenia-related status in community-dwelling older adults.

**Methods:**

Study participants were 269 older adults living in rural Japan. A bioelectrical impedance analysis device was used to measure body composition parameters including body mass index (BMI), body fat percentage, waist-hip-ratio, and appendicular skeletal muscle mass index (SMI). Muscle function was measured by handgrip strength and 6-m walking speed. The correlation coefficients for C3 level and measurements were calculated using Pearson correlation analysis. Participants were categorized into normal, pre-sarcopenia, dynapenia, or sarcopenia groups. Sarcopenia was defined according to 2019 Asian Working Group for Sarcopenia definition, dynapenia was defined as low muscle function without low muscle mass, and pre-sarcopenia was defined as the presence of low muscle mass only. The C3 threshold score for sarcopenia status was evaluated by *receiver operating characteristic curve* (ROC) analysis.

**Results:**

Significant positive correlations were found between C3 and BMI, body fat percentage, and waist-hip ratio in both sexes, and further positive correlations with SMI were found in women. The relationship with body fat percentage was particularly strong. Body composition measurements (BMI, body fat percentage, and waist- hip ratio) and C3 levels were lowest in the sarcopenia group compared with the others. ROC analysis showed that the significant threshold of C3 for discriminating between the normal and sarcopenia groups was 105 mg/dL. Multiple logistic regression analysis showed that participants with C3 < 105 mg/dL had an odds ratio of 3.27 (95% confidence interval, 1.49–7.18) for sarcopenia adjusted by sex, age and body fat percentage.

**Conclusion:**

C3 levels are suggested to be related to body composition and pathophysiological functions of sarcopenia. C3 is expected to become a useful biomarker for sarcopenia, for predicting the onset of the disease and for predicting the effectiveness of interventions.

## Background

In Japan, the population of elderly people is 36.23 million people, accounting for 29.1% of the total population, the highest ever, and it is expected to continue to increase [1]. Sarcopenia is a geriatric syndrome characterized by progressive and generalized loss of muscle mass and strength, with risk of physical disability, reduced quality of life, and increased rate of mortality [2–4]. As the elderly population increases, not only in Japan but also worldwide, the number of people with sarcopenia is rapidly increasing, and countermeasures are urgently required.

Diagnostic methods for sarcopenia are currently based on objective body composition and function. Meanwhile, no specific biomarker candidates for sarcopenia have yet been identified. If the risk of sarcopenia can be assessed at an early stage using biomarkers that can predict the onset and the effectiveness of interventions (e.g., exercise therapy and nutritional supplementation), and if drug therapy can be appropriately determined, the onset of sarcopenia may be delayed and preventive care can be promoted [5]. In a 2017 systematic review, pooled analysis of 16 cross-sectional studies showed that blood C-reactive protein (CRP) was significantly higher in people with sarcopenia than in people without sarcopenia, although there was high heterogeneity across the studies. Conversely, IL-6 and TNF1α were not significantly different between people with and without sarcopenia [6]. Thus, no specific biomarker candidates have yet been identified for sarcopenia.

Meanwhile, the complement system is a complex immune network, and recent advances have led to its recognition as an antimicrobial system and as a diverse regulator of immunity and tissue homeostasis [7]. C3 complement in particular plays an important role in management of the complement system. The relationship between C3 and pathological conditions has been reported within the relationship between serum C3 levels and cardiometabolic diseases, such as hypertension, atherosclerosis and fatty liver [8, 9]. In addition, C3 has been associated with obesity and body fat in young and middle-aged people [10, 11].

C3 is produced not only in the liver, adipose tissue and macrophages [12], it is also produced in human myoblasts [13, 14]. Complement synthesis by myocytes is upregulated by inflammatory cytokines such as INF-γ and IL-1β [13], suggesting that C3 plays an important role in the local production and activation of complements under inflammatory pathophysiological conditions in muscle tissue. High levels of C3, then, may therefore have a protective effect against inflammatory pathophysiology in muscle tissues, leading to the prevention against muscle loss. The potential role of C3 in mitigating inflammatory processes within muscle tissues has therefore been established, but there are gaps in the understanding of how variations in C3 levels specifically correlate with body composition and with the pathology of sarcopenia among older adults in community settings. The present study seeks to determine the relationship between serum complement C3 levels and body composition and sarcopenia-related status in community-dwelling older adults.

## Methods

### Participants

**Fig. 1:**
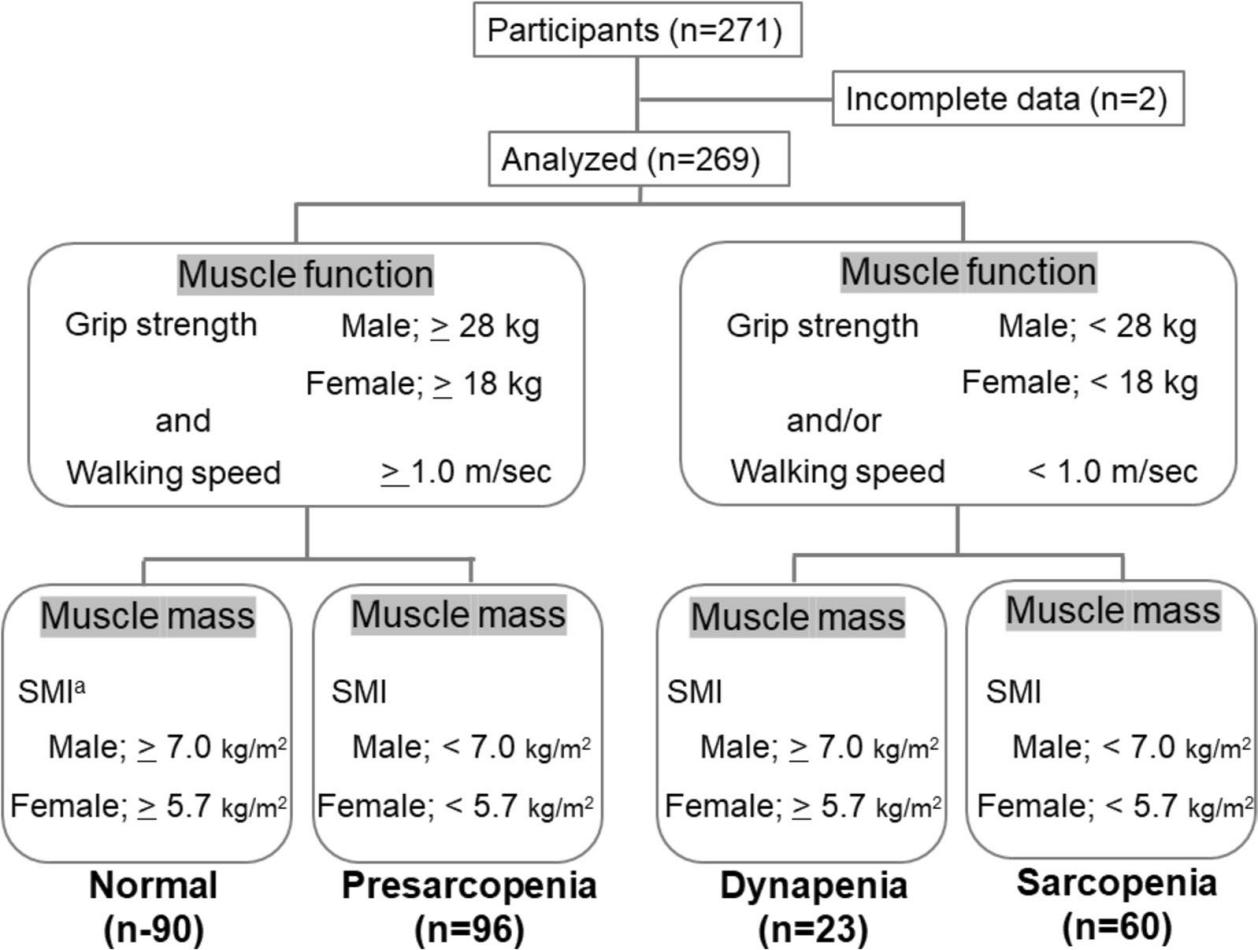
Assessment of normal, pre-sarcopenia, dynapenia, and sarcopenia. ^a^skeletal muscle mass index

This was a cross-sectional, population-based study conducted in Kaizuka City, Osaka Prefecture, Japan, between 2020 and 2022. All participants underwent an annual health checkup co-sponsored by the Kaizuka City Elderly Care Division and Osaka Kawasaki Rehabilitation University. The inclusion criteria were age ≥ 60 years, living independently at home, and not having a cardiac pacemaker. In total, 269 participants were analyzed, after exclusion of two patients who had incomplete data (Fig. 1). The calculated sample size required for a four-group comparison in a cross-sectional study was 232 (effect size, 0.25; α error, 0.05; power, 0.9), so the actual sample size of 269 was thought to be appropriate. This study was approved by the Osaka Kawasaki Rehabilitation University Ethics Committee (Reference No. OKRU-RA0020) and was performed in accordance with the Declaration of Helsinki. Written informed consent was obtained from all participants before the study began.

### Measurements of body composition

Body composition parameters were measured using a bioelectrical impedance analysis (BIA) device (InBody 270; InBody, Tokyo, Japan) at 20 and 1000 kHz. Participants wore normal indoor clothing without shoes or socks [15]. They were instructed to grasp the handles of the BIA device and stand on electrodes contacting the bottoms of their feet. We measured body mass index (BMI), appendicular skeletal muscle mass index (SMI), body fat percentage, and waist-to-hip ratio. BMI was calculated as bodyweight in kilograms divided by height in meters squared (kg/m^2^). SMI was calculated as muscle mass in kilograms divided by height in meters squared (kg/m^2^). Body fat percentage was calculated as body fat weight divided by bodyweight.

### Measurements of muscle function

Handgrip strength was measured with a grip dynamometer (T.K.K. 5401, Takei Scientific Instruments Co., Ltd., Niigata, Japan). Measurements were taken with the grip dynamometer in an upright position with the dominant hand adjusted so that the second joint of the index finger was at a 90° angle, and the arm naturally lowered. Gait speed was expressed as the average of speed of five trials of 6 m normal walking starting without acceleration and ending without deceleration.

### Evaluation of sarcopenia

Sarcopenia was defined in this study as the presence of low muscle mass plus reduced muscle strength indicated as decreased hand grip and/or slow walking speed, according to the 2019 Asian Working Group for Sarcopenia (AWGS) criteria [16]. Low muscle mass was defined as height-adjusted SMI *<* 7.0 kg/m^2^ for men and *<*5.7 kg/m^2^ for women. Decreased hand grip was defined as handgrip strength *<* 28 kg for men and *<*18 kg for women, and slowness was defined as a 6-m usual gait speed *<* 1.0 m/s. Dynapenia was defined as low muscle function without low muscle mass [17], and pre-sarcopenia was defined as the presence of low muscle mass only [2] (Fig. 1).

### Serum biochemical measurements

All participants fasted for 2 h before blood collection, and blood samples were drawn between 10:00 and 15:00. Complement C3 and albumin levels were measured. Blood analyses were performed at a laboratory within 24 h of collection (Japan Clinical Laboratories, Inc., Kyoto, Japan). Total C3 and albumin levels were measured by immunoturbidimetry and bromocresol purple method, respectively.

### Statistical analysis

The correlation coefficients for serum substances and age, and body composition were calculated using Pearson correlation analysis. Participants were categorized into normal, pre-sarcopenia, dynapenia, or sarcopenia groups. The significance of differences among groups was assessed by one-way analysis of variance. Comparisons between groups were made by Student’s t-test. To examine the influence of body composition on C3 level, we conducted multiple regression analysis adjusted by sex and age. The C3 threshold score for discriminating the status of sarcopenia was evaluated by *receiver operating characteristic curve* (ROC) analysis. In this analysis, 1-specificity and sensitivity are calculated for each C3 level that distinguishes between normal and sarcopenia, plotted on a plane with 1-specificity on the horizontal axis and sensitivity on the vertical axis, and connected with lines to create an ROC curve. The cutoff value was defined as the point with the minimum distance to the upper left corner. The AUC value is the area under the ROC curve. Odds ratios of C3 for sarcopenia status were calculated using multiple logistic regression analyses. C3, sex, age, BMI and body fat percentage were used as independent variables, and sarcopenia as the dependent variable. Statistical analysis was conducted using JMP 17 (SAS Institute, Cary, NC). All statistical tests were two-tailed, and a significance level of 0.05 was used.

## Results

Age, body compositions, each measure of physical performance, serum albumin and C3, and the status of sarcopenia are shown in Table 1. Ninety-six participants (35.69%) had pre-sarcopenia, 23 participants had dynapenia (8.55%), and 60 participants (22.30%) had sarcopenia.

**Table 1:** Characteristics of the study participants

	All	Men	Women
Number	269	63	206
Age (years)	74.50 (6.51)^a^	75.29 (6.57)	74.26 (6.49)
BMI^b^ (kg/m^2^)	22.30 (3.05)	23.59 (2.89)	21.91 (3.00)
Body fat percentage (%)	30.21(6.80)	25.70 (6.51)	31.58 (6.29)
Waist-hip ratio	0.82(0.06)	0.84 (0.07)	0.81 (0.05)
SMI^c^ (kg/m^2^)	5.90 (1.00)	7.28 (0.08)	5.48 (0.62)
Grip strength (kg)	22.87 (7.05)	31.76 (6.79)	20.15 (4.38)
Walking speed (m/sec)	1.31 (0.21)	1.26 (0.23)	1.32 (0.20)
Albumin (g/dL)	4.14 (0.27)	4.13 (0.24)	4.15 (0.28)
C3 (mg/dL)	104.36 (18.14)	98.90 (17.05)	106.03 (18.17)
Pre-sarcopenia	96 (35.69)^d^	16 (25.40)	80 (38.83)
Dynapenia	23 (8.55)	10 (15.87)	13 (6.31)
Sarcopenia	60 (22.30)	8 (12.69)	52 (25.24)

^a^mean (standard deviation): ^b^body mass index: ^c^smooth muscle mass index: ^d^number (%)

**Fig. 2:**
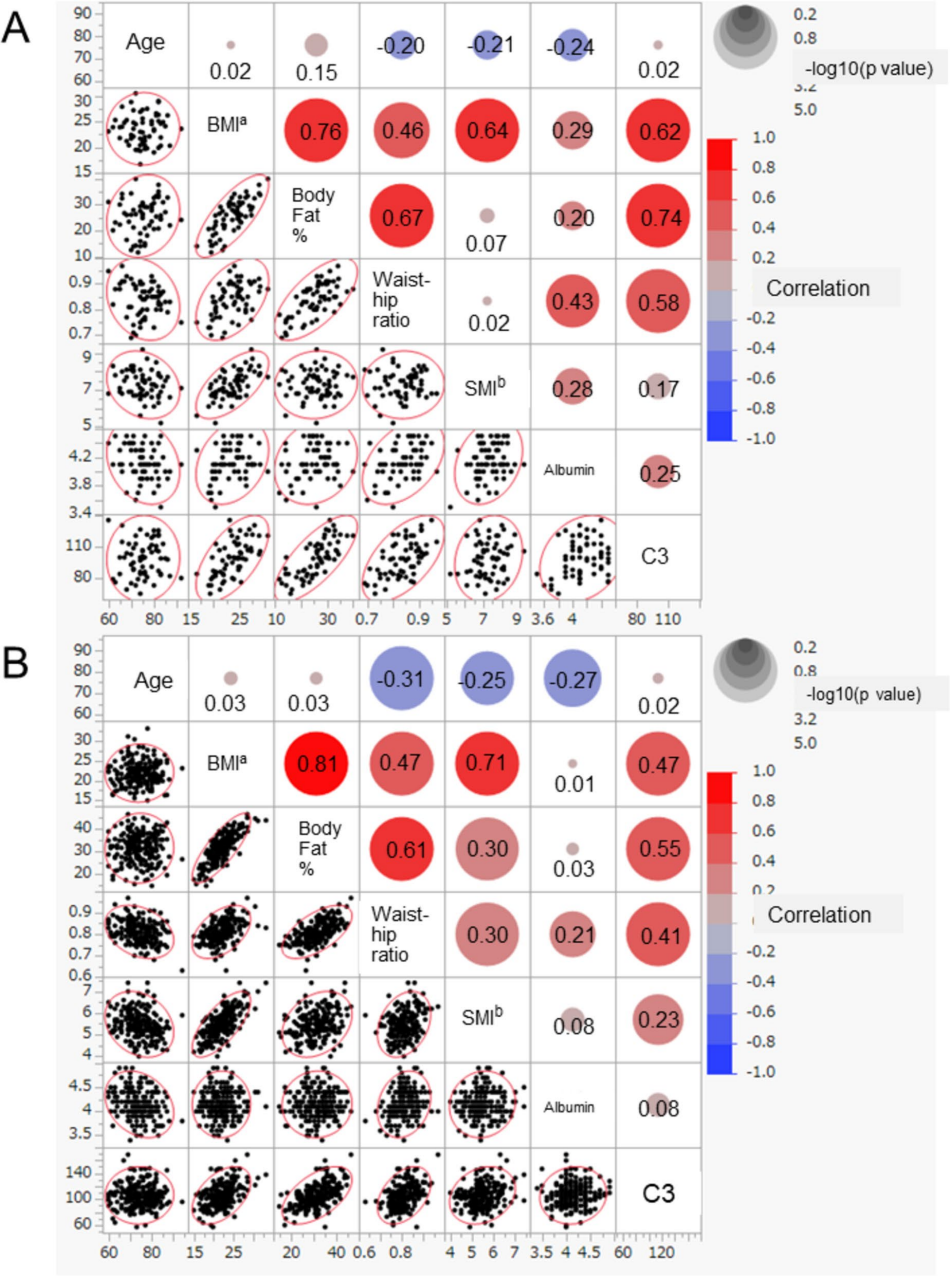
Scatterplot matrix of age, body composition, and serum substances in men (**A**) and women (**B**). Scatterplots between age, BMI, body fat percentage, waist-hip ratio, SMI, albumin, and C3 are shown on the lower triangular part. The red circle shows probability ellipse. Circle size indicates significance, and the color of a circle shows correlation on the upper triangular part. ^a^body mass index; ^b^smooth muscle mass index

**Fig. 3:**
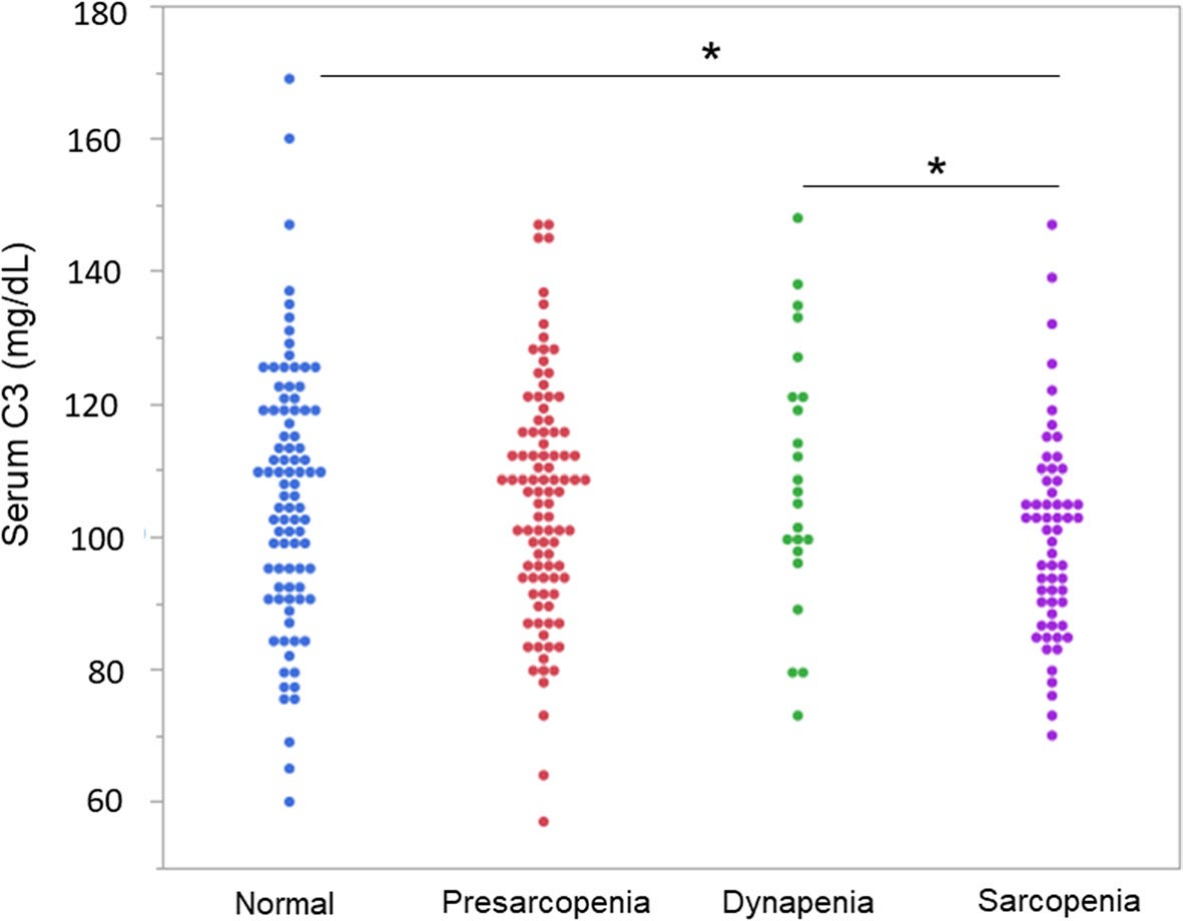
Scatterplot of C3 level in normal, pre-sarcopenia, dynapenia, and sarcopenia. *P < 0.05

Regarding C3 and body composition by sex, a significant positive correlation was found between C3 and BMI (r = 0.616, *P* < 0.0001), body fat percentage (r = 0.744, *P* < 0.0001) and waist-hip ratio (r = 0.580, *P* < 0.0001) in men. In women, significant positive correlations were found between C3 and BMI (r = 0.472, *P* < 0.0001), body fat percentage (r = 0.554, *P* < 0.0001), waist-hip ratio (r = 0.414, *P* < 0.0001), and SMI (r = 0.232, *P* = 0.0008). No correlations between C3 and age were found for either sex (Fig. 2).Comparing body composition in the normal, pre-sarcopenia, dynapenia and sarcopenia groups, the results showed that BMI, body fat percentage, and waist-hip ratio were lower in the sarcopenia group than in the other groups (Table 2). There was also a significant reduction in C3 in the sarcopenia group compared with the normal group and the dynapenia group (normal vs sarcopenia; mean = 105.90 vs 99.67 mg/dL, *P* = 0.0485, dynapenia vs sarcopenia; 108.43 vs 99.67 mg/dL, *P* = 0.0391) (Table 2) (Fig. 3). Multiple regression analysis of each body composition for C3 adjusted by sex and age showed that standard partial regression coefficient (β) of percent body fat had the highest value (β =0.506; *P* < 0.0001) (Table 3).

**Table 2:** Comparison of characteristics between normal, pre-sarcopenia, dynapenia, and sarcopenia

	normal	pre-sarcopenia	dynapenia	sarcopenia	*P*
Sex Men Women	29 (46.0) 61(29.6)	16 (25.4) 80 (38.8)	10 (15.9) 13 (20.6)	8 (12.7) 52 (25.2)	0.0019
Age (years)	72.11(5.75)	74.13(6.52)	77.57(7.13)	77.50(5.78)	< 0.0001
BMI^b^ (kg/m^2^)	23.87(2.74)	21.14(2.25)	25.73(2.85)	20.50(2.44)	< 0.0001
Body fat percentage (%)	30.15(7.44)	30.01(6.24)	32.12(7.36)	29.86(6.50)	0.5633
Waist-hip ratio	0.84(0.06)	0.81(0.05)	0.81(0.06)	0.80(0.05)	< 0.0001
SMI^c^ (kg/m^2^)	6.64(0.87)	5.50(0.60)	6.86(0.94)	5.07(0.62)	< 0.0001
Grip strength (kg)	27.61(7.02)	23.25(4.60)	19.55(6.63)	16.41(4.41)	< 0.0001
Walking speed (m/sec)	1.36(0.17)	1.36(0.18)	1.19(0.20)	1.19(0.23)	< 0.0001
Albumin (g/dL)	4.21(0.27)	4.15(0.28)	4.10(0.25)	4.06(0.26)	0.0114
C3 (mg/dL)	105.90(19.55)	104.88(17.67)	108.43(19.60)	99.67(15.42)	0.0775

^a^mean (standard deviation): ^b^body mass index: ^c^smooth muscle mass index

**Table 3:** Multiple regression analysis for body composition predicting serum C3 level

	*β* ^b^	*P*	VIF^c^
Body fat percentage	0.506	< 0.0001	2.183
Waist-hip ratio	0.156	0.0248	2.061
SMI^a^	0.128	0.1047	2.674

All data were adjusted by sex and age

^a^smooth muscle mass index; ^b^standard partial regression coefficient; ^c^variance inflation factor

ROC analysis showed that the threshold of C3 level corresponding to identify the normal and the sarcopenia groups was 105.00 mg/dL (AUC = 0.608, sensitivity; 71.67%, specificity; 52.15%, *P* = 0.0206). No significant cutoff values were detected for pre-sarcopenia and dynapenia groups (Table 4). Multiple logistic regression analysis showed that participants with C3 < 105 mg/dL had an OR of 3.31 (95% confidence interval [CI] 1.62-6.73; *P* = 0.0010), 2.24 (95% CI 1.05-4.79; *P* = 0.0372), and 3.27 (95% CI 1.49-7.18; *P* = 0.0031) for sarcopenia adjusted by age and sex (Model 1), by age, sex and BMI (Model 2), and by age, sex and body fat percentage (Model 3), respectively (Table 5).

**Table 4:** Threshold values of C3 level for pre-sarcopenia, dynapenia, and sarcopenia

	Threshold values of C3 (mg/dL)	AUC^a^	Sensitivity (%)	Specificity (%)	*P*
Pre-sarcopenia	106.00	0.521	52.08	56.07	0.7286
dynapenia	96.00	0.564	82.61	33.74	0.2640
sarcopenia	105.00	0.608	71.67	52.15	0.0206

^a^AUC area under the curve

**Table 5:** Multiple logistic regression analysis for odds ratio of the C3 < 105 mg/dL for sarcopenia

	Odds ratio	95%CI^a^	*P*
Model 1	3.31	1.62–6.73	0.0010
Model 2	2.24	1.05–4.79	0.0372
Model 3	3.27	1.49–7.18	0.0031

^a^CI: confidence interval

Model 1, adjusted by sex and age; Model 2, adjusted by sex, age and BMI; Model 3, adjusted by sex, age and body fat percentage

## Discussion

C3 levels or C3 are suggested to be related to body composition and pathophysiological functions of sarcopenia.

- Relationship between C3 levels and body composition:

**Serum C3 levels showed associations with BMI, body fat percentage, waist-hip ratio, and SMI in older adults.** Among these, the relationship with body fat percentage was particularly strong. A study of young adults with body weight in the reference range reported significantly higher C3 in the group with high body fat percentage compared with the group with low body fat percentage [11]. C3 was previously reported to be positively associated with BMI in a longitudinal study of patients in middle age, in which an increase in C3 over 7 years was positively associated with an increase in BMI [10]. Our results are thought to be particularly novel in that they show that the relationship between C3 and fat mass also appeared in older adults.

(b) Associations between C3 and sarcopenia:

**Our results suggest that complement C3 plays a role in mitigating the development of sarcopenia in older adults living in the community by reducing inflammation and promoting muscle regeneration.** Our findings demonstrate significant links between complement C3 levels and sarcopenia in this demographic. Notably, individuals diagnosed with sarcopenia exhibited lower C3 levels compared to those without this condition. We also identified correlations between C3 and various body composition parameters, including BMI, body fat percentage, and waist-hip ratio. Interestingly, our result indicates that decreased C3 levels, falling below the threshold of 105.00 mg/dL, are associated with a higher risk of sarcopenia, even after adjustments for age, sex, BMI, and body fat percentage. These findings support complement C3’s potential role in reducing the onset of sarcopenia among older adults, likely due to its anti-inflammatory properties and support for muscle regeneration processes.

**Fig. 4:**
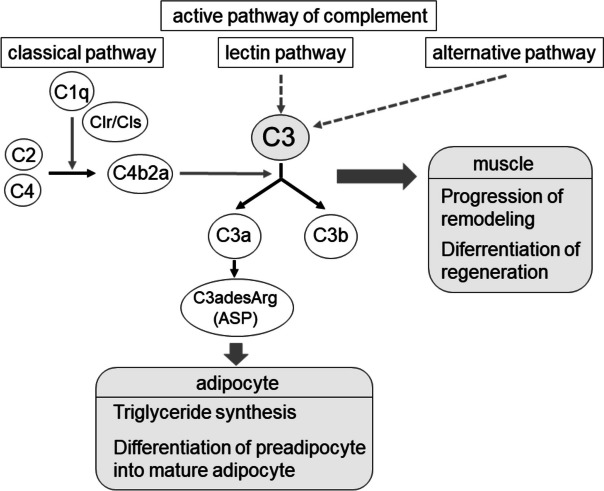
Relationship between C3 and adipose tissue and muscle tissue in the complement activation pathway [13, 14, 18–23, 29, 32]. ASP, acylation stimulating protein

(c) Complement pathway and adipocyte biology:

**The observed relationship between C3 levels and the body fat parameters epidemiologically supports biochemical studies on the roles played by the active complement pathway involving C3 and acylation stimulating protein in glucose transporter transmission, triglyceride synthesis, and adipocyte differentiation.** The active pathway of complement consists of three pathways, each with a different activator: the classical pathway (CP), the alternative pathway and the lectin pathway, and their subsequent convergent pathways. In CP, complement Clq forms a complex with complement Clr/Cls, which degrades complement C4 and complement C2 to form C4b2a (C3 convertase), and then C4b2a degrades C3 to C3a and C3b. C3 transferases produced from all three pathways also further activate C3 [18]. The intermediate product of this pathway, C3a, is cleaved to produce C3adesArg (also known as acylation stimulating protein; ASP) [19]. ASP is an adipocyte-derived protein with potent anabolic effects on human adipose tissue, it transmits glucose transporters from intracellular sites to the cell surface, activates diacylglycerol acyltransferase (DGAT), and promotes triglyceride synthesis in adipocytes [20–23] (Fig. 4).

Furthermore, C3 gene-deficient mice (which are deficient in C3 protein) have been observed to have less weight gain than mice with the C3 gene [24–26]. C3-C3a-ASP axis is suggested by these reports to be related to adipocyte biology and total body weight (Fig. 4). Our results strongly support the findings of these reports.

(d) The role of C3 in myogenic cell differentiation and muscle remodeling:

**The observed relationship between C3 levels and SMI epidemiologically supports biochemical studies on there being a potential role for C3 in muscle remodeling.** Myogenic cell differentiation is a complex process involving the activation, proliferation and differentiation of progenitors [27–30]. In studies of mouse fetal muscle cells, proteomic and transcriptomic analyses have reported that C3 promotes the differentiation of myogenic progenitors following internalization of immune molecules [31]. Expression of C3 is also found in human myoblasts [13]. These effects strongly suggest that C3 is involved in the progression of muscle remodeling and differentiation of regenerative parts [32], and support our findings of a relationship between SMI and C3 (Fig. 4).

The increased complement C1q level in sarcopenia has been reported [33] and it has been identified as a biomarker for the development of frailty and age-related diseases [34, 35]. When the complement system is activated, a series of reactions involving proteolysis and assembly occur, resulting in cleavage of C3. These results suggest that in sarcopenia, an increase in C1q in CP may activate the classical pathway in peripheral blood, increasing C3 activation and cleavage and reducing its levels (Fig. 4).

(e) Study limitations

The current study has a small number of limitations. First, all participants were recruited from one location and the data therefore only represent results from one area. Confirmation of our results requires replication of the findings from other geographic locations from a larger number of subjects. Second, the number of men was small. It is hoped that the relationship between C3 and body composition by sex will become clearer in the future by increasing the number of men analyzed. Thirdly, both AWGS and the European Working Group on Sarcopenia in Older People propose the use of SMI BIA measurements to assess muscle mass, but the gold standards for muscle mass measurement are computed tomography and magnetic resonance imaging, so the use of BIA has several drawbacks. Finally, this study included patients with inflammatory diseases, so future analyzes will need to exclude patients with these diseases.

## Conclusion

This is the first Japan-based report on the relationship between sarcopenia and C3 in community-dwelling older adults. Low serum C3 levels were associated with low body composition indices such as BMI, body fat percentage, and waist-hip ratio, as well as with sarcopenia. C3 levels are suggested to be related to the determination of body composition and pathophysiological functions of sarcopenia. The findings obtained in this study are expected to contribute to the elucidation of the onset mechanism of sarcopenia and risk assessment using C3 as a biomarker, and are expected to lead to the advancement of sarcopenia research and the future development of prevention methods.

## Data Availability

The data are available from the corresponding author upon reasonable request.

## Abbreviations

AUC: Area under the curve
AWGS: Asian Working Group for Sarcopenia
BIA: Bioelectrical impedance analysis
BMI: Body mass index
CI: Confidence interval
ROC analysis: Receiver operating characteristic analysis
SMI: Smooth muscle mass index
